# NADPH–Cytochrome P450 Reductase Mediates the Fatty Acid Desaturation of ω3 and ω6 Desaturases from *Mortierella alpina*

**DOI:** 10.3390/cimb44050125

**Published:** 2022-04-22

**Authors:** Mingxuan Wang, Jing Li, Wenjie Cong, Jianguo Zhang

**Affiliations:** Institute of Food Science and Engineering, School of Health Science and Engineering, University of Shanghai for Science and Technology, Shanghai 200093, China; mxwang567@163.com (M.W.); usstlj@163.com (J.L.); congwj_0204@163.com (W.C.)

**Keywords:** ω3 desaturase, ω6 desaturase, NADPH–cytochrome P450 reductase, enzyme kinetics, *Mortierella alpina*

## Abstract

Fatty acid desaturases play an important role in maintaining the appropriate structure and function of biological membranes. The biochemical characterization of integral membrane desaturases, particularly ω3 and ω6 desaturases, has been limited by technical difficulties relating to the acquisition of large quantities of purified proteins, and by the fact that functional activities of these proteins were only tested in an NADH-initiated reaction system. The main aim of this study was to reconstitute an NADPH-dependent reaction system in vitro and investigate the kinetic properties of *Mortierella alpina* ω3 and ω6 desaturases in this system. After expression and purification of the soluble catalytic domain of NADPH–cytochrome P450 reductase, the NADPH-dependent fatty acid desaturation was reconstituted for the first time in a system containing NADPH, NADPH–cytochrome P450 reductase, cytochrome b5, *M. alpina* ω3 and ω6 desaturase and detergent. In this system, the maximum activity of ω3 and ω6 desaturase was 213.4 ± 9.0 nmol min^−1^ mg^−1^ and 10.0 ± 0.5 nmol min^−1^ mg^−1^, respectively. The highest *k*_cat_/*K*_m_ value of ω3 and ω6 desaturase was 0.41 µM^−1^ min^−1^ and 0.09 µM^−1^ min^−1^ when using linoleoyl CoA (18:2 ω6) and oleoyl CoA (18:1 ω9) as substrates, respectively. *M. alpina* ω3 and ω6 desaturases were capable of using NADPH as reductant when mediated by NADPH–cytochrome P450 reductase; although, their efficiency is distinguishable from NADH-dependent desaturation. These results provide insights into the mechanisms underlying ω3 and ω6 fatty acid desaturation and may facilitate the production of important fatty acids in *M. alpina*.

## 1. Introduction

Fatty acid desaturases convert saturated fatty acids into unsaturated fatty acids. These enzymes are present in all groups of organisms and play a key role in the functionality of biological membranes; they also help to prevent inflammatory diseases [1,2,3]. Each fatty acid desaturase introduces an unsaturated bond at a specific position in a fatty acyl chain, and exhibits remarkable capability for the regional and stereo-selective introduction of unsaturated bonds [4]. Gaining a better understanding of the molecular mechanisms and biochemical properties of fatty acid desaturases has become a key objective in scientific research.

There are two types of fatty acid desaturases, as determined by solubility: the soluble acyl-ACP desaturases that are found in the plastids of higher plants, and the integral membrane desaturases that are found in the endomembrane systems of prokaryotes and eukaryotes [5,6]. ω3 desaturase and ω6 desaturase are membrane-type desaturases that perform important functions by converting fatty acids into ω3 and ω6 fatty acids that are essential for human life, including arachidonic acid (ARA; 20:4 ω6), eicosapentaenoic acid (EPA; 20:5 ω3), and docosahexaenoic acid (DHA; 22:6 ω3) [7,8]. *M. alpina* is an oleaginous fungus that can produce a number of lipids in excess of 50% of its own dry weight. This fungus produces EPA via the ω3 PUFA biosynthetic pathway when cultured below 20 °C, and produces ARA via the ω6 PUFA biosynthetic pathway [9,10]. In the previous study, active forms of *M. alpina* ω6 and ω3 desaturase were expressed and purified in sufficient amounts to allow biochemical characterization, and the activity of these enzymes was determined via coupling of two electron-transport proteins: cytochrome b5 (Cytb5) and NADH–cytochrome b5 reductase (NADH-Cytb5R) [11].

**Figure 1:**
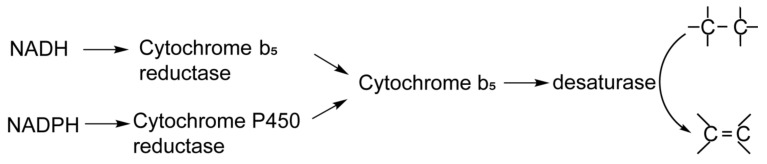
Schematic showing the two-electron transport systems.

It is known that the endoplasmic reticulum of eukaryotic cells contains two electron transfer systems [12,13]. These systems are provided with electrons by two different flavoprotein reductases, an FAD-containing enzyme, NADH-Cytb5R [14], and an FAD- and FMN-containing enzyme, NADPH–cytochrome P450 reductase (NADPH-CytP450R) (Figure 1) [15]. Nicotinamide adenine dinucleotide phosphate (NADPH) and nicotinamide adenine dinucleotide (NADH) are the sources of electrons that reduce the enzymes CytP450R and Cytb5R, respectively. Previous reports have shown that NADPH can serve as the reductant for the desaturation of stearyl-CoA and that the resultant activity was similar to that obtained in the NADH-dependent system [16]. It was also possible to reconstitute NADPH-dependent linoleoyl-CoA desaturation in a system containing NADPH-CytP450R; in this situation, the desaturase activity was only 60% of that exhibited by NADH-dependent desaturation [17]. However, both the stearyl-CoA desaturase and linoleoyl-CoA desaturase mentioned above were derived from rat liver. The ω3 and ω6 desaturases genes were lost during evolution in mammals, and conserved domains differ in some desaturases from mammals and microorganisms; for example, the fused cytochrome b5-like domain in fungal Δ9 desaturase is absent in mammalian stearyl-CoA desaturase [18]. Thus, differences in the electron transport system, between mammalian and fungal desaturation, may exist, and whether the *M. alpina* ω3 and ω6 desaturation could also use NADPH as a reductant, and how efficient the ω3 and ω6 desaturases function with different substrates, remains unknown.

In the present study, to reconstitute ω3 and ω6 desaturation in the NADPH-initiated system, firstly, the soluble catalytic domain of CytP450R was expressed in *Escherichia coli* and purified in soluble form. Then, after verifying that this protein was able to catalyze the NADPH-dependent reduction of Cytb5, the ω3 and ω6 desaturation activity was confirmed in a system containing ω3 and ω6 desaturase, CytP450R, Cytb5, and detergent. Finally, by exploiting this system, desaturation activities and kinetic data were determined using different substrates and the desaturation efficiency was compared with NADH-dependent desaturation. To our knowledge, these are the first kinetic data of membrane-type ω3 and ω6 desaturases derived from an NADPH-CytP450R-mediated electron transport system. The investigation of NADPH-depend ω3 and ω6 fatty acid desaturation may provide insights into their desaturation mechanism and guide the industrial application of the oleaginous fungus *M. alpina*.

## 2. Materials and Methods

### 2.1. Expression and Purification of NADPH-CytP450R and Cytb5

The codon-optimized gene for human NADPH-CytP450R was synthesized by GenScript, and sequenced and subcloned into the pET15b vector with 6 histidine tags on the 5′ end of the target gene using *Nde*I and *Bam*HI sites. The plasmid was then transformed into *E. coli* BL21(DE3) cells and cultured in LB media containing 100 μM riboflavin and 100 mg/L ampicillin. Protein expression was induced using 0.4 mM IPTG when the OD_600_ of the culture reached 0.6–0.8. The induction was performed at 25 °C for 24 h. To purify the NADPH-CytP450R protein, cells were harvested and resuspended in lysis buffer (20 mM HEPES pH 7.9, 500 mM KCl, 5 mM imidazole, 10% glycerol, 0.1% Triton X-100, 40 μg/mL Dnase I, 1 mM MgCl2, and 0.1 mM of PMSF and benzamidine protease inhibitors) and lysed by sonication. After centrifugation at 12,000 rpm for 25 min, the supernatant was loaded onto a column containing Cobalt Resin (Thermo Fisher Scientific, Waltham, MA, USA). Then, three column volumes of buffer were used to wash the column, and a linear gradient of 5–500 mM imidazole was used to elute the protein. Fractions containing NADPH-CytP450R were pooled. The protein solution was then dialyzed overnight against 25 mM Tris buffer (pH 8.0) and then concentrated using a Vivaspin concentrator. The Cytb5 protein was prepared according to a published method [11]. The cells were resuspended and lysed. The protein was firstly purified using HisPur Cobalt Resin (Thermo Scientific) column. Fractions containing the protein of interest were pooled and dialyzed against 20 mM Tris pH 8.0 overnight. Then, protein was purified from the dialysate using a Q-Sepharose FF column (GE Healthcare, Milwaukee, WI, USA). The final step of the purification involved separation on the HiLoad 16/60 Superdex 200 column equilibrated with 20 mM Hepes pH 7.5, 100 mM NaCl. The protein solution was concentrated using a Vivaspin concentrator. The His-tag was removed from Cytb5 by the addition of 0.2 U/mg of biotinylated thrombin during the dialysis step; the thrombin was removed by incubation with streptavidin agarose beads. For Cytb5, a 3-molar excess of hemin chloride, was added and incubated for 1.5 h prior to injection of the Superdex 200 column, in order to ensure full loading of this critical cofactor.

### 2.2. Assays to Determine the Activity of NADPH-CytP450R and Cytb5 Proteins

The enzymatic activities of NADPH-CytP450R and Cytb5 were determined by monitoring the absorption features of Cytb5. Assays were performed in triplicate using a Quartz cuvette and a Beckman DU800 spectrophotometer. The 800 µL reaction mixture contained 100 mM Hepes pH 7.5, 150 mM NaCl, 0.25 µM purified CytP450R, 5 µM purified Cytb5, and 5µM NADPH. The enzyme mixture (0.25 µM purified CytP450R and 5 µM purified Cytb5, 100 mM HEPES pH 7.5, 150 mM NaCl) and NADPH were incubated separately at 25 °C for 5 min. The incubated materials were then mixed, and the absorption features were monitored at 25 °C. All experiments were performed three times.

### 2.3. Preparation and Kinetic Analysis of ω3 and ω6 Desaturases in an NADPH-Initiated System

*M. alpina* ATCC 32222 was purchased from the American Type Culture Collection (Manassas, VA, USA). The desaturase-coding sequences were identified from *M. alpina* ATCC 32222, and the *M. alpina* ω3 and ω6 desaturases were prepared as previously described [11]. Briefly, the ω3 and ω6 desaturase genes were appended to a cassette containing the human rhinovirus 3C protease cleavage site, the IgG-specific ZZ-tag, and an RGS-His10-tag, cloned into a pPink-HC vector (Invitrogen) and expressed in *Pichia pastoris*. After cell lysis, the membrane fractions containing desaturases were separated using an ultracentrifuge and the desaturases were efficiently extracted using 1% (*w*/*v*) Fos-Choline-16 solution. The solubilized membranes were incubated overnight at 4 °C with IgG Sepharose 6 Fast Flow (GE Healthcare, Milwaukee, WI, US). The IgG Sepharose was then washed three times at 4 °C with 30 resin volumes of wash buffer. Desaturases were released from the IgG Sepharose by treatment with His-tagged rhinovirus 3C protease. The His-tagged protease was then removed by incubation for 2 h at 4 °C with HisPur Cobalt Superflow Agarose (Thermo Scientific, Waltham, MA, USA). The desaturases were then concentrated and subjected to size exclusion chromatography on a HiLoad 16/60 Superdex 200 column (GE Healthcare, Milwaukee, WI, USA). The column was eluted with buffer at a flow rate of 1 mL/min. Fractions containing the desaturase, based on SDS-PAGE analysis, were concentrated, and prepared for activity assay.

The activity of *M. alpina* ω3 desaturase and ω6 desaturase in an NADPH-initiated system were determined according to the method described previously, with modifications [11]. The activity was determined by monitoring the reoxidation of Cytb5 in the presence and absence of fatty acid-CoA substrates at 422 nm and 28 °C. Assays were performed in triplicate on three separate days using 96-well UV-transparent half-area microplates (Corning) and a SpectraMax 340PC384 absorbance microplate reader. The reaction system contained 25 mM Hepes pH 7.5, 1 µM NADPH, 150 mM NaCl, 100 nM desaturase, 0.5 µM Cytb5, 1 µM CytP450R, 0.002% Fos-16, and 0–300 µM fatty acid-CoA substrate. The Cytb5 remains reduced until all the NADPH was consumed, and then Cytb5 became oxidized. The desaturase activity was calculated by determining the difference between the time for NADPH oxidation in the absence and presence of fatty acid-CoA, and it was assumed that 1 mol of NADH was required for the formation of 1 mol of the unsaturated fatty acid-CoA product. The *k*_cat_, *K*_m_, and *k*_cat_/*K*_m_ values were analyzed using non-linear regression in GraphPad Prism. Reaction products were verified by GC/MS analysis of fatty acid methyl esters [19].

### 2.4. Nucleotide Sequence Accession Number

The codon-optimized nucleotide sequence of human a NADPH–cytochrome P450 reductase gene was deposited in the GenBank database under the accession number MZ667610. The codon-optimized nucleotide sequence of Cytb5 gene was deposited in the GenBank database under the accession number MF101850.

## 3. Results

### 3.1. Purification of NADPH-CytP450R and the Reduction of Cytb5 by NADPH-CytP450R

**Figure 2:**
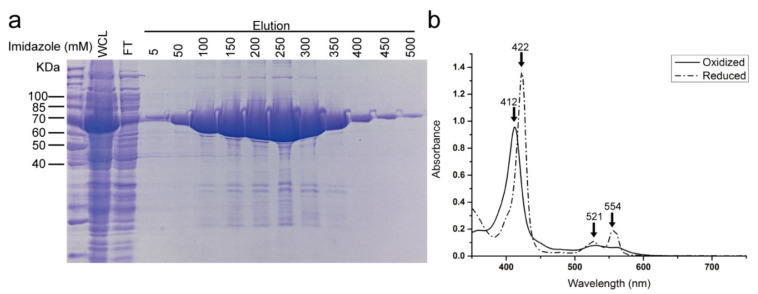
Purification of soluble CytP450R and the reduction of Cytb5 by CytP450R. (**a**) SDS-PAGE analysis of whole cell lysate (WCL), flow-through (FT) and elution fractions collected during cobalt affinity purification. (**b**) Wavelength scan of oxidized and reduced Cytb5 by CytP450R.

Cytochrome P450 reductase is a membrane-bound enzyme that is required for the transfer of electrons from NADPH to cytochrome P450 and Cytb5 in the endoplasmic reticulum of eukaryotic cells. In order to obtain a soluble NADPH-CytP450R recombinant protein, the soluble catalytic domain was fused with a 6-histidine tag and expressed in *E. coli* (Figure S1 in Supplementary Materials) [20]. After induction with IPTG, prominent bands were observed at approximately 70 kDa in the supernatant of cell lysates, thus indicating the successful expression of human soluble CytP450R (Figure S1 in Supplementary Materials). During the purification, the CytP450R protein bound efficiently to the cobalt resin and was eluted with gradient imidazole solution. The resulting protein was homogenous and consistent with its theoretical molecular weight, as shown in Figure 2A. The purified Cytb5 was also homogenous and fully loaded with heme (Figure S2 in Supplementary Materials). To determine the activity of CytP450R and to verify the coupling of CytP450R and Cytb5, we monitored the absorbance features of Cytb5 in the absence and presence of NADPH. Results showed that the oxidized Cytb5 exhibited strong absorption at 412 nm; however, when NADPH was added, Cytb5 was instantly reduced and the absorbance features shifted to 422, 521, and 554 nm (Figure 2B).

### 3.2. ω3 and ω6 Fatty Acid-CoA Desaturation in the NADPH-CytP450R-Mediated Reaction System

**Figure 3:**
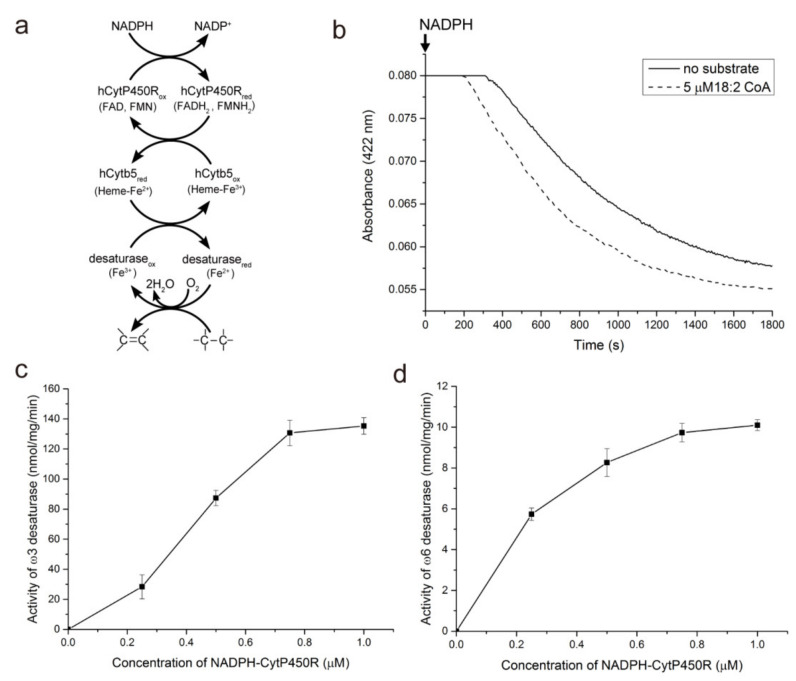
The principle of electron transport and dependence of NADPH-initiated desaturase activity on the concentration of cytochrome P450 reductase in the reconstituted system: (**a**) The reaction scheme used to monitor desaturase activity. NADPH and human cytochrome P450 reductase (hCytP450R) were used to rapidly reduce human cytochrome b5 (hCytb5) and in turn the desaturase. (**b**) Representative progression curves for the reaction between ω3 desaturase and 18:2 CoA substrate. Note that the re-oxidation of Cytb5 was accelerated by the addition of substrate versus air oxidation in the blank. (**c**) Dependence of ω3 desaturase activity on the concentration of CytP450R. (**d**) Dependence of ω6 desaturase activity on the concentration of CytP450R.

After verifying the coupling of the electron-transport proteins CytP450R and Cytb5, the ω3 and ω6 desaturation were reconstituted in the NADPH-initiated reaction system. The ω3 and ω6 fatty acid desaturase were purified as homogenous and active proteins (Figure S3 in Supplementary Materials). Figure 3A illustrates the principle of electron transfer from NADPH to the terminal ω3 and ω6 desaturase. Upon the addition of NADPH, the cofactors for CytP450R were reduced, followed subsequently by the Heme-Fe^3+^ in Cytb5. When the di-iron in desaturase was reduced, the fatty acid substrates could be desaturated in the presence of oxygen. Thus, by monitoring the reduction and re-oxidation of Cytb5 at 422 nm, the time difference between the presence and absence of substrates enabled the calculation of desaturase activity (Figure 3B). Next, we optimized the amount of CytP450R to be added to the system in order to obtain the highest desaturase activity in the NADPH-initiated reaction system. As shown in Figure 3C,D, the desaturation activity of ω3 and ω6 desaturase was dependent on the amount of CytP450R present. When the concentration of CytP450R increased from 0.25 to 0.75 µM, the activity of ω3 desaturase increased rapidly. The maximum activity reached 138.9 ± 7.8 nmol min^−1^ mg^−1^ when 1 µM CytP450R was added. The activity of ω6 desaturase also showed a positive correlation with the concentration of CytP450R and the highest activity was achieved when 1 µM CytP450R was added.

### 3.3. Kinetic Analysis of ω3 and ω6 Desaturases in NADPH-Dependent Desaturation

**Figure 4:**
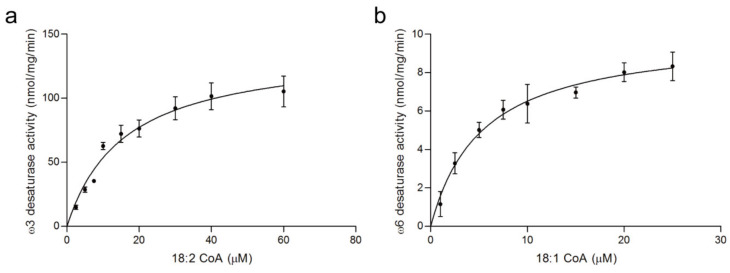
Kinetic analysis of NADPH-dependent ω3 and ω6 desaturation. (**a**) Michaelis–Menten analysis of the reaction between ω3 desaturase and 18:2-CoA. (**b**) Michaelis–Menten analysis of the reaction between ω6 desaturase and 18:1-CoA. See Table 1 for kinetic values determined for these substrates and others.

By exploiting this NADPH-initiated system, we were able to determine the activities of *M. alpina* ω6 desaturase and ω3 desaturase against a panel of fatty acid substrates (Figure 4 and Figure S4 in Supplementary Materials, Table 1). Results showed that the maximum activity of ω6 desaturase was 10.0 ± 0.5 nmol min^−1^ mg^−1^ using oleoyl CoA (18:1 ω9) as substrate, which was 53.2% of the activity observed in NADH-initiated reaction assay (Table S1 in Supplementary Materials) [11]. The maximum activity of ω3 desaturase was 213.4 ± 9.0 nmol min^−1^ mg^−1^ using linoleoyl CoA (18:2 ω6) as substrate, which was 59.0% of the activity observed in NADH-initiated reaction assay. In the NADPH-dependent system, the *k*_cat_/*K*_m_ value of *M. alpina* ω6 desaturase for oleoyl-CoA was approximately twofold greater than for pamitoleoyl-CoA, indicating that oleoyl-CoA is the preferred substrate. The ω3 desaturase also showed marked differences for linoleoyl, γ-linolenoyl, and arachidonoyl-CoA substrates in the NADPH-initiated system. The highest *k*_cat_/*K*_m_ value of ω3 desaturase was 0.41 µM^−1^ min^−1^ when using linoleoyl CoA (18:2 ω6) as substrates. While the *K*_m_ value for γ-linolenoyl-CoA was approximately sixfold higher than that for linoleoyl-CoA, the highest *K*_m_ value of 157 ± 23.8 µM was observed for arachidonoyl-CoA. These data suggested that ω3 and ω6 desaturases are capable of using NADPH as a reductant, and the best substrates for ω3 and ω6 desaturases were linoleoyl-CoA and oleoyl- CoA, respectively.

## 4. Discussion

Fatty acid desaturases perform important functions and generate a variety of unsaturated and polyunsaturated fatty acids. ω6 desaturase introduces a double bond into oleic acid to form the first ω6 fatty acid, while ω3 desaturase can potentially convert all ω6 fatty acids into corresponding ω3 fatty acids [21]. Extensive functional studies of desaturase genes have been carried out previously in vivo via heterologous expression in *Saccharomyces cerevisiae* [22,23,24]. However, these studies did not involve the detailed biochemical analysis of highly purified membrane desaturase. This was due to difficulties in obtaining sufficient quantities of purified desaturases. Previous studies of microsomal stearyl-coenzyme A desaturase from rat liver revealed that two other purified proteins, NADH–cytochrome b5 reductase and cytochrome b5, are required for the transport of electrons in the microsomal stearyl–CoA desaturation system [16,25]. In the previous study, human soluble NADH–cytochrome b5 reductase and cytochrome b5 were coupled to construct a desaturation system, and detailed kinetic analyses of *M. alpina* ω3 desaturase and ω6 desaturase were performed [11].

The other source of electrons, NADPH, is known to be a key cofactor that is required for the synthesis and desaturation of fatty acids in oleaginous microbes [26,27,28]. In this study, the soluble catalytic domain of human NADPH-CytP450R was expressed and purified. This soluble catalytic domain is the C-terminal 614 residues of the membrane-bound intact protein (residues VRESSFV through SLDVWS*), which contained the conserved domain CYPOR and Flavodoxin_1 essential for catalytic activity (Figure S1c in Supplementary Materials), and the results of gene sequencing revealed no mutations on the gene sequence of this soluble catalytic domain. The interaction of CytP450R with purified Cytb5 demonstrated that the pure CytP450R was capable of reducing Cytb5 in the presence of NADPH, thus suggesting that NADPH could be used for ω3 and ω6 fatty acid desaturation in micelles. A previous study has reported that the activity of desaturase was dependent on the amount of reductase [17]; however, in this study, after providing a sufficient amount of CytP450R, the NADPH-dependent desaturation was not as efficient as that produced by NADH-dependent desaturation when using the same amounts of the terminal enzyme. This led us to hypothesize that this difference is presumably related to the difference in electron transfer efficiency of the two systems. However, when Enoch and Strittmatter suggested that NADPH might be the actual physiological reductant for fatty acid desaturation under certain metabolic conditions [12], the difference observed in the two electron systems in vitro might also be caused by the environment for these enzymes, such as micelles and lipid environment, considering NADPH-CytP450R, NADH-Cytb5R, and Cytb5 are amphipathic proteins under natural conditions. This indicated that the coupling of these proteins is not as efficient as or may differ from the endogenous, membrane-associated reduction system. Since the three-dimensional structure of ω3 and ω6 desaturase and their complex with cofactors have not been solved to date, continued investigations are needed to determine how the three proteins interact in the different reactions described above. Nevertheless, these results lead us to conclude that the NADH-dependent system might be more preferable for the ω3 and ω6 desaturation in the oleaginous *M. alpina*.

The oleaginous fungus *M. alpina* can produce both ω3 and ω6 fatty acids and has been used on an industrial scale to produce ARA (20:4 ω6). Previous studies implied that fatty acid synthesis was possibly affected by NADPH generated by cytosolic enzymes and that fatty acid desaturation was affected by NADPH produced by membrane-bound enzymes [29]. Furthermore, *M. alpina* has been metabolically engineered for enhanced ARA production by improving the supply of NADPH [30]. However, the production of the more desirable ω3 fatty acids, particularly long-chain polyunsaturated EPA (20:5 ω3), is still under investigation. Based on the results of this research, a supply of NADH could be more advantageous for the ω3 fatty acid desaturation.

## 5. Conclusions

In this study, the ω3 and ω6 desaturation was reconstituted in an NADPH-dependent system for the first time. The *M. alpina* ω3 and ω6 desaturases were capable of using NADPH as reductant when mediated by NADPH-CytP50R; although, their efficiency is distinguishable from NADH-dependent desaturation in vitro. These results provide insights into the mechanisms underlying ω3 and ω6 fatty acid desaturation and may facilitate the production of important fatty acids in *M. alpina*.

## Figures and Tables

**Table 1:** Kinetic parameters for *M. alpina* ω6 and ω3 desaturases with different fatty acid-CoA substrates in NADPH-dependent desaturation.

Desaturase	Substrate	Specific Activity (nmol min^−1^ mg^−1^)	*K*_m_ (µM)	*k*_cat_ (min^−1^)	*k*_cat_/*K*_m_ (µM^−1^ min^−1^)
ω6	18:1 ω9	10.0 ± 0.5 ^a^	5.4 ± 0.8	0.5 ± 0.02	0.09
ω6	16:1 ω7	3.5 ± 0.2	3.9 ± 0.9	0.2 ± 0.01	0.04
ω3	18:2 ω6	138.9 ± 7.8	16.0 ± 2.2	6.6 ± 0.6	0.41
ω3	18:3 ω6	213.4 ± 9.0	87.8 ± 9.9	10.1 ± 0.4	0.12
ω3	20:4 ω6	28.8 ± 1.3	157.0 ± 23.8	1.4 ± 0.1	0.01

^a^ Standard deviation.

## Data Availability

Data are contained within the article or Supplementary Materials.

## Supplementary Materials

The following supporting information can be downloaded at: https://www.mdpi.com/article/10.3390/cimb44050125/s1, Figure S1: Expression and purification of soluble CytP450R. (a) Map of the expression vector pET15b-Cytochrome P450 reductase. (b) Comparison of CytP450R *E. coli* transformants before and after IPTG induction by SDS-PAGE analysis. (c) Conserved domains on the soluble catalytic domain of human NADPH-cytochrome P450 reductase; Figure S2. Purification of Cytb5. (a) SDS-PAGE analysis of whole cell lysate (WCL), flow-through (FT) and elution fractions collected during cobalt affinity purification. (b) and (c) SDS-PAGE analysis of ion exchange purification using Q-Sepharose FF column. (d) SDS-PAGE analysis of gel filtration fractions and concentrated protein (P); Figure S3. Purification of ω3 desaturase. (a) SDS-PAGE analysis of IgG affinity purification. Lane 1: Sample loaded; Lane 2: Flow through; Lane 3: Protein bound to IgG beads; Lane 4–6: standard BSA; Lane 7: Protein bound to IgG beads. (b) Release of ω3 desaturase from IgG affinity column. Lane 1–4: Elution of IgG column; Lane 5: After incubation with cobalt beads; Lane 6–8: standard BSA; Lane 9: IgG beads after elution. (c) Size exclusion of concentrated sample. ω3 desaturase bound to IgG beads was indicated by black arrow. HRV 3C Protease was indicated by red arrow; Figure S4. Kinetic analysis of NADPH-dependent ω3 and ω6 desaturation. (a) Michaelis-Menten analysis of the reaction between ω3 desaturase and 18:3-CoA. (b) Michaelis-Menten analysis of the reaction between ω3 desaturase and 20:4-CoA. (c) Michaelis-Menten analysis of the reaction between ω6 desaturase and 16:1-CoA. See Table 1 for kinetic values determined for these substrates; Table S1: Kinetic parameters for *M. alpina* ω6 and ω3 desaturases with various fatty acid-CoA substrates in NADH-dependent desaturation.
