# Supplementary material for: NADPH–Cytochrome P450 Reductase Mediates the Fatty Acid Desaturation of ω3 and ω6 Desaturases from Mortierella alpina
Source: Curr Issues Mol Biol. 2022 Apr 22;44(5):1828–37. doi: 10.3390/cimb44050125 (PMC9164069; doi:10.3390/cimb44050125)
Supplement: Supplementary file 1 [file cimb-44-00125-s001.zip › cimb-1672500-supplementary.pdf]

Supplementary information for

NADPH-cytochrome P450 reductase mediates the fatty acid desaturation of ω3 and ω6 desaturases from *Mortierella alpina*

Mingxuan Wang[1], Jing Li[1], Wenjie Cong[1], Jianguo Zhang[1*]

[1]Institute of Food Science and Engineering, School of Health Science and Engineering, University of Shanghai for Science and Technology, Shanghai 200093, China

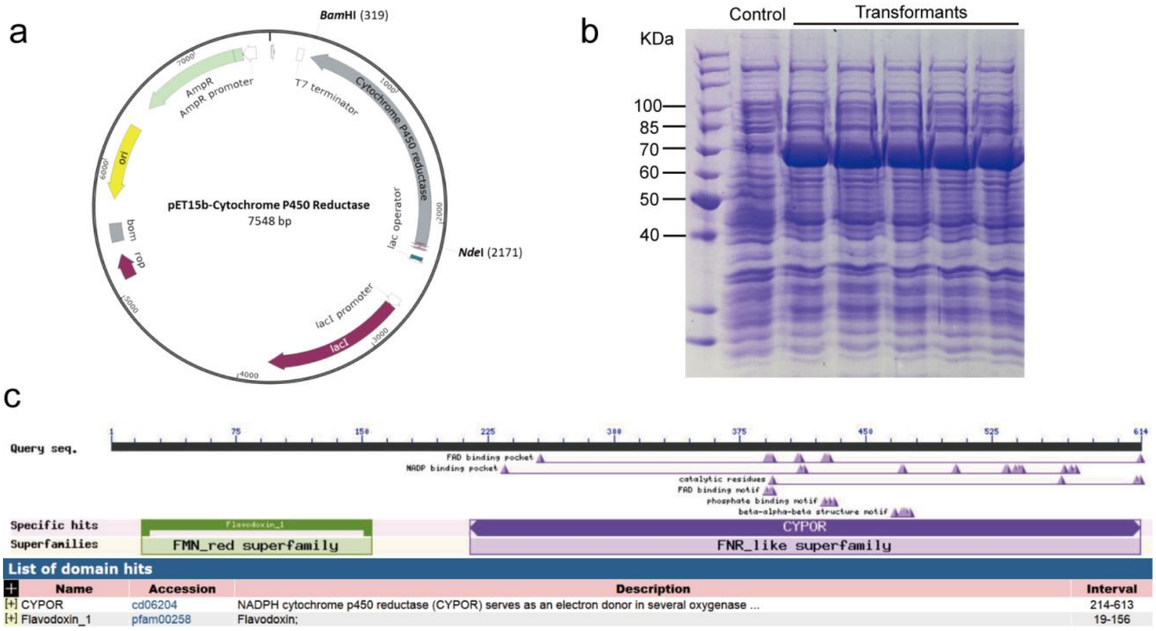

Figure S1. Expression and purification of soluble CytP450R. (a) Map of the expression vector pET15b-Cytochrome P450 reductase. (b) Comparison of CytP450R *E.coli* transformants before and after IPTG induction by SDS-PAGE analysis. (c) Conserved domains on the soluble catalytic domain of human NADPH-cytochrome P450 reductase.

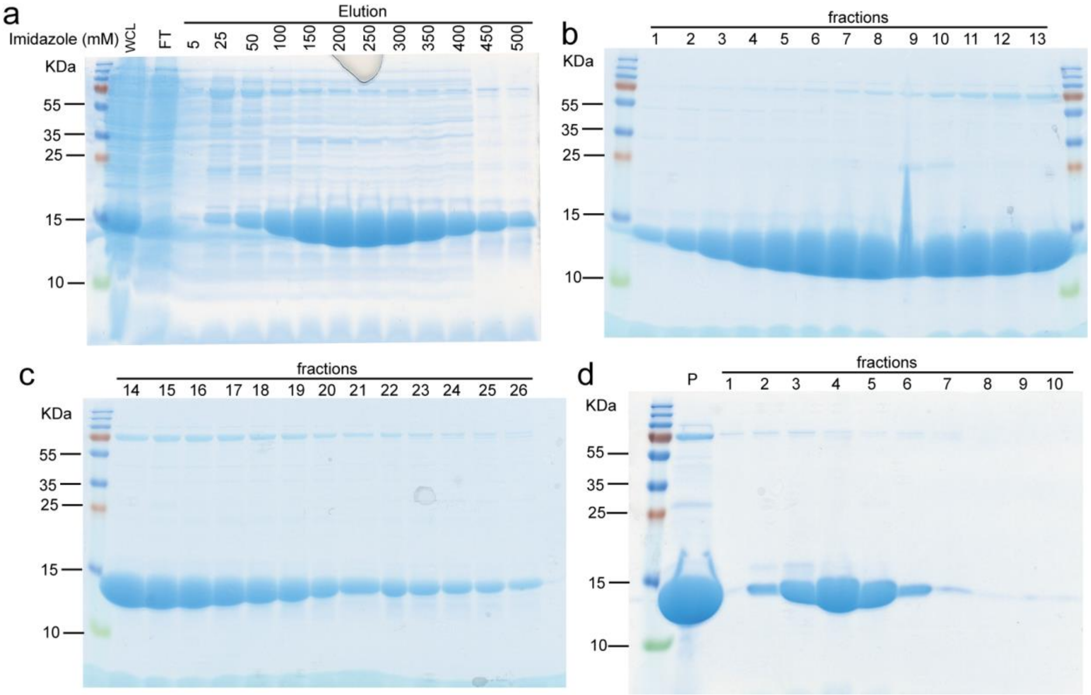

Figure S2. Purification of Cytb5. (a) SDS-PAGE analysis of whole cell lysate (WCL), flow-through (FT) and elution fractions collected during cobalt affinity purification. (b) and (c) SDS-PAGE analysis of ion exchange purification using Q-Sepharose FF column. (d) SDS-PAGE analysis of gel filtration fractions and concentrated protein (P).

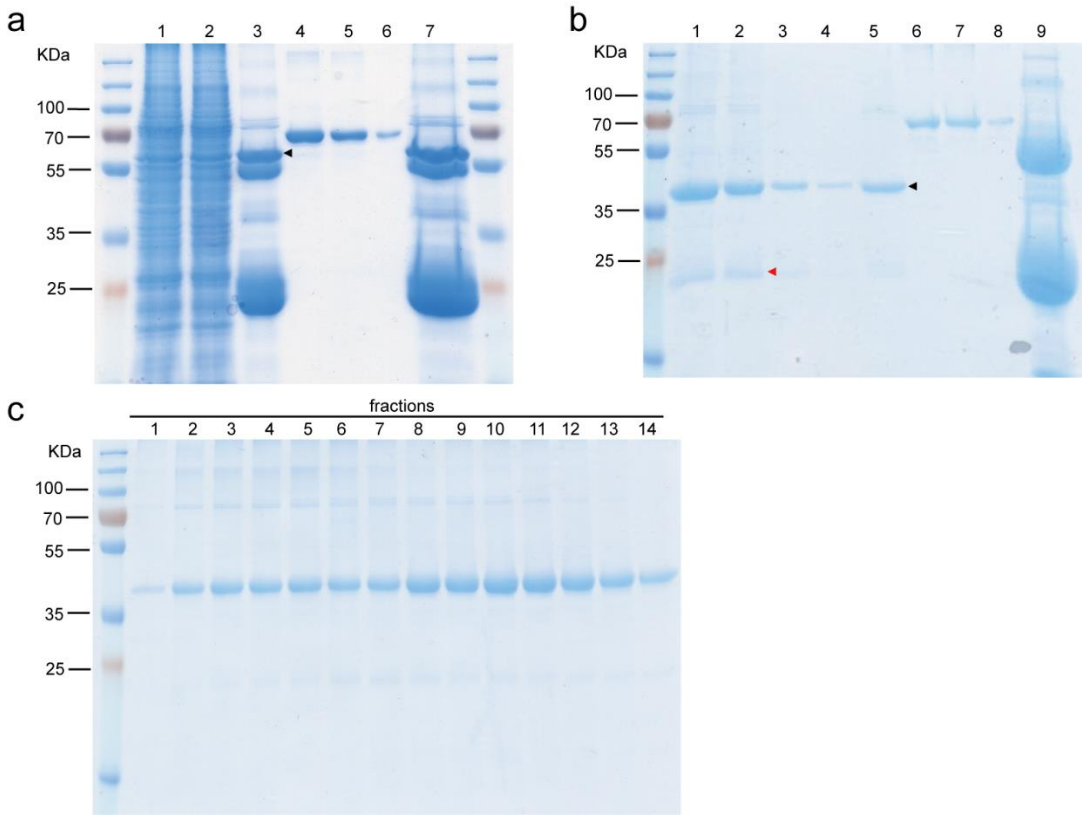

Figure S3. Purification of ω3 desaturase. (a) SDS-PAGE analysis of IgG affinity purification. Lane1: Sample loaded; Lane2: Flow through; Lane3: Protein bound to IgG beads; Lane4-6: standard BSA; Lane7: Protein bound to IgG beads. (b) Release of ω3 desaturase from IgG affinity column. Lane1-4: Elution of IgG column; Lane 5: After incubation with cobalt beads ; Lane 6-8: standard BSA; Lane 9: IgG beads after elution. (c) Size exclusion of concentrated sample. ω3 desaturase bound to IgG beads was indicated by black arrow. HRV 3C Protease was indicated by red arrow.

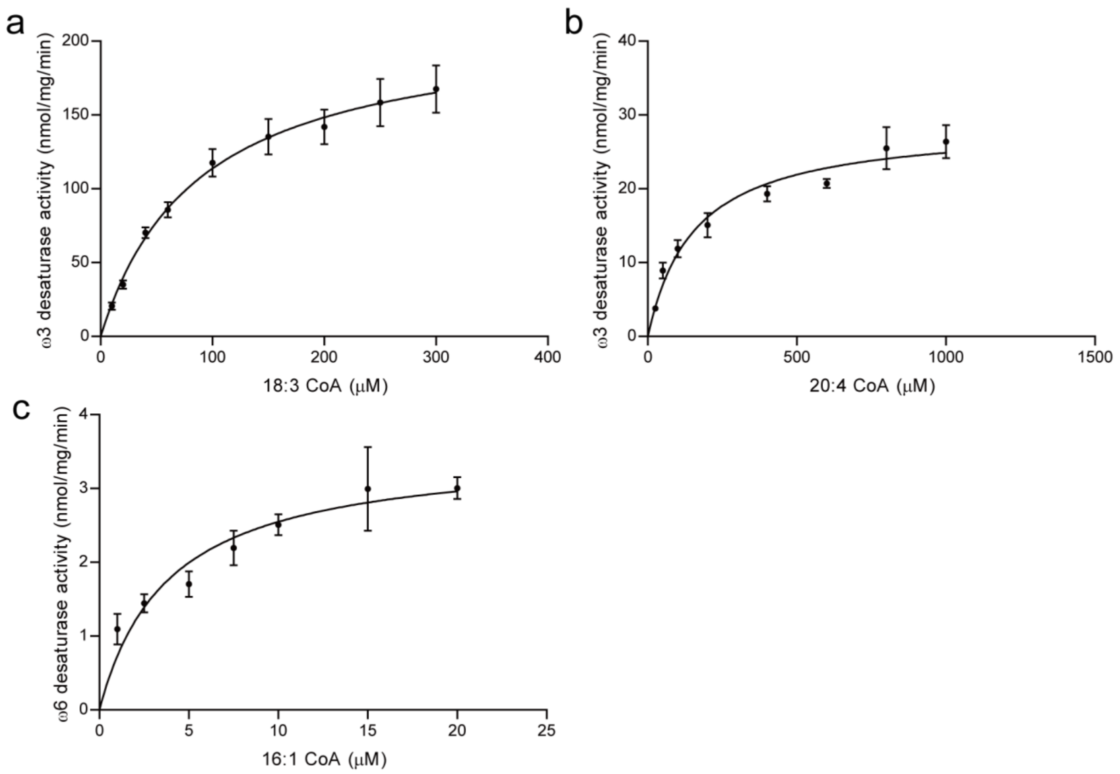

Figure S4. Kinetic analysis of NADPH-dependent ω3 and ω6 desaturation. (a) Michaelis-Menten analysis of the reaction between ω3 desaturase and 18:3-CoA. (b) Michaelis-Menten analysis of the reaction between ω3 desaturase and 20:4-CoA. (c) Michaelis-Menten analysis of the reaction between ω6 desaturase and 16:1-CoA. See Table 1 for kinetic values determined for these substrates.

Table S1. Kinetic parameters for *M. alpina* ω6 and ω3 desaturases with various fatty acid-CoA substrates in NADH-dependent desaturation [11].

Desaturase	Substrate	Specific Activity (nmol min^{-1} mg^{-1})	K_m (μM)	k_{cat} (min^{-1})	k_{cat}/K_m ($μM^{-1}$ min^{-1})
ω6	18:1 *n*-9	18.8 ± 0.9[a]	5.4 ± 0.8	0.9 ± 0.04	0.16
ω6	16:1 *n*-7	6.0 ± 0.4	3.7 ± 0.8	0.3 ± 0.02	0.09
ω3	18:2 *n*-6	235.3 ± 13.2	15.9 ± 2.2	11.2 ± 0.6	0.70
ω3	18:3 *n*-6	464.6 ± 19.5	87.9 ± 9.9	22.0 ± 0.9	0.25
ω3	20:4 *n*-6	57.2 ± 2.5	157.4 ± 23.9	2.7 ± 0.1	0.02

[a]standard deviation.
